# „Die Enkel der Osmanen“ – Ausgrenzung, Diskriminierung und Illegalisierung von „Tagelöhnern“ im Münchner Bahnhofsviertel

**DOI:** 10.1007/s41059-021-00082-5

**Published:** 2021-03-09

**Authors:** Kaan Atanisev, Rita Haverkamp

**Affiliations:** Stiftungsprofessur für Kriminalprävention und Risikomanagement, Eberhard Karls Universität Tübingen, Geschwister-Scholl-Platz, 72074 Tübingen, Deutschland

## Abstract

Im Münchner Bahnhofsviertel gibt es eine „Tagelöhnerbörse“, die viel Unmut unter Anwohnenden und Gewerbetreibenden hervorruft. Die überwiegend aus Bulgarien stammenden „Tagelöhner“ sind dabei vielfältigen Ausgrenzungs- und Diskriminierungserfahrungen ausgesetzt, die zum Teil aus ihrer Angehörigkeit zu einer ethnischen Minderheit resultieren. Bei näherer Betrachtung zeigt sich allerdings, dass ihre ethnische Zugehörigkeit komplexer und vielschichtiger ist, als man auf den ersten Blick erahnt. Im Beitrag steht die Frage nach der Rolle der ethnischen Zugehörigkeit der „Tagelöhner“ im Mittelpunkt, um anhand ethnografischer Feldgespräche und Interviews die Aushandlungsprozesse von Zugehörigkeit im multiethnischen Bahnhofsviertel nachzuzeichnen. Nach einer Beschreibung der „Tagelöhner“ im Münchner Bahnhofsviertel wird die soziokulturelle Lage der ethnischen Minderheiten in Bulgarien dargestellt. Aus der von Uneindeutigkeit charakterisierten Zugehörigkeit der „Tagelöhner“ ergeben sich sowohl Vor- als auch Nachteile im Umgang mit den Gewerbetreibenden und Anwohnenden des Bahnhofsviertels. Abschließend wird die Illegalisierung der „Tagelöhnerbörse“ und deren Auswirkungen auf die Stellung der „Tagelöhner“ diskutiert.

## Abstract (en)

In the Munich train station district there is a “day laborer market” that is much resented by residents and tradespeople. The predominantly Bulgarian “day laborers” face various experiences of exclusion and discrimination, partly due to their affiliation to an ethnic minority. A closer look shows that their ethnicity is more complex and multi-layered than one might guess at first. This article focuses on the role of ethnicity of “day laborers” in order to illustrate, by means of ethnographic field talks and interviews, the negotiation processes of belonging in the multi-ethnic train station district. After describing the “day laborers” in the train station district, the socio-cultural situation of ethnic minorities in Bulgaria is addressed. The “day laborers” affiliation is ambiguous and results, thus, in both advantages and disadvantages in dealing with residents and tradespeople of the train station district. Finally, the illegalization of the “day laborer” market and its effects on their position are discussed.

## Einleitung

Anfang der 2000er Jahre etablierte sich im südlichen Münchner Bahnhofsviertel eine „Tagelöhnerbörse“, die erstmals im Jahr 2013 in der lokalen Medienberichterstattung negative Aufmerksamkeit erhielt. Seither sind die Stadt München und die Münchner Polizei mit diesem an einer engen Kreuzung konzentrierten Arbeitsmarkt befasst, der den dort ansässigen Gewerbetreibenden ein Dorn im Auge ist und immer wieder Beschwerden ihrerseits hervorruft (Landeshauptstadt München 2019). Die „Tagelöhner“ selbst fühlen sich einerseits von ihrem Umfeld stigmatisiert und andererseits von der Polizei kriminalisiert. Im August 2019 erhöhte sich der bestehende Verdrängungsdruck für die „Tagelöhner“ durch eine Gesetzesänderung im Schwarzarbeitsgesetz. Das Gesetz enthält nun ein Verbot von Arbeitsmärkten im öffentlichen Raum und sieht die Anordnung von vorübergehenden Platzverweisen und Betretungsverboten vor. Neben Razzien durch den Zoll und Polizeikontrollen gibt es auch verschiedene soziale Hilfsangebote. Speziell für „Tagelöhner“^1^ eröffnete die Stadt im Jahr 2015 ein Beratungscafé in der Nähe des Arbeitsmarktes. Bereits im Jahr 2010 nahm die nichtstaatliche Initiative für Zivilcourage e. V. ihre Tätigkeit auf und unterstützt seither „Tagelöhner“ bei Konflikten mit Behörden, der Polizei und Arbeitgebenden (Initiative Zivilcourage München 2016).

Im März 2020 brachte die Covid-19-Pandemie den Arbeitsmarkt zum Erliegen. Daraufhin kehrten die meisten „Tagelöhner“ in ihre Heimat zurück und wurden dort im Vergleich zur Mehrheitsbevölkerung mit verschärften Restriktionen unter Quarantäne gestellt. Die Betroffenen wurden im Frühjahr und Sommer oft in ihren Stadtvierteln oder Dörfern eingepfercht und durften diese nur unter strengen Kontrollen verlassen, so dass ihnen der Zugang zu außerhalb liegenden Apotheken weitgehend verwehrt und die Versorgung mit Medikamenten nicht gewährleistet war. Die in München verbliebenen Menschen waren vollständig vom dortigen Unterstützungsnetz abhängig, das für sie in eingeschränkterem Umfang Essen, Kleidung, Übernachtung und medizinische Versorgung bereithielt.

Der Beitrag skizziert zunächst das methodische Vorgehen. Anschließend geht es um eine Beschreibung der Situation der „Tagelöhner“ im südlichen Münchner Bahnhofsviertel, und danach um Zugehörigkeiten, die unter der Überschrift „ethnische Grenzziehungen“ unter Berücksichtigung der Situation der ethnischen Minderheiten in Bulgarien thematisiert werden. Mit Diskriminierung und Ausgrenzung sind die „Tagelöhner“ sowohl in Bulgarien als auch in Deutschland konfrontiert. Während diese in Bulgarien in Armut, im Bildungsbereich und in abgeschotteten Wohnvierteln zum Ausdruck kommt, ist hierzulande ihr Leben auf das Nötigste und auf die Arbeitssuche im öffentlichen Raum reduziert. Im Münchner Bahnhofsviertel sind sie nicht nur Zollrazzien und Polizeikontrollen ausgesetzt, sondern auch dem Gutdünken der Arbeitgebenden, die ihnen den Lohn vorenthalten bzw. den Mindestlohn unterschreiten und die Anmeldung und Abführung der Beiträge von sozialversicherungspflichtigen Beschäftigungen unterlassen.

## Methodisches Vorgehen

Der vorliegende Beitrag beruht auf einem empirischen Ausschnitt aus dem vom Bundesministerium für Bildung und Forschung geförderten Verbundprojekt „Sicherheit im Bahnhofsviertel (SiBa)“.^2^ Die Datenbasis für diesen Beitrag bilden dabei 15 Interviews mit Gewerbetreibenden (u. a. Hotels, Supermärkte, Gastronomie) sowie Sozialarbeitenden im Münchner Bahnhofsviertel (u. a. Wohnungslosen- und Drogenhilfe, Streetwork, Beratungsdienste für Arbeitssuchende) sowie ethnografische Feldprotokolle. Bei der Durchführung kamen qualitative Leitfadeninterviews zum Einsatz (Przyborski und Wohlrab-Sahr 2014, S. 126 ff.), die neben narrativen Elementen auch eine Auswahl an Themenbereichen beinhalteten, die auf Basis der Erkenntnisse einer vorangegangenen quantitativen Bewohnendenbefragung gebildet wurden. Dies ermöglichte es, Schwerpunkte im Leitfadeninterview zu setzen und im Anschluss erste theoretische Vorannahmen zu formulieren. So zeigte sich in der quantitativen Befragung, dass im Münchner Bahnhofsviertel das Vertrauen in die Hilfsbereitschaft der Nachbarschaft (44 % gegenüber 77 %) und das Vertrauen insgesamt in die Nachbarschaft (66 % gegenüber 89 %) weitaus geringer ausfällt als in anderen Stadtteilen.^3^ Dies warf generelle Fragen über das Ausmaß des Nachbarschaftsgefühls und der sozialen Kontrolle im Bahnhofsviertel auf. Unter sozialer Kontrolle sind hierbei alle sozialen Strukturen, Mechanismen und Prozesse gemeint, die abweichendes Verhalten verhindern oder einschränken sollen (Meier 2010, S. 224); dies kann informell (z. B. Familie und Nachbarschaft) und formell (z. B. Polizei) durch positive oder negative Reaktionen auf konformes oder abweichendes Verhalten geschehen (Lamnek 2018, S. 317; Kunz und Singelnstein 2016, S. 253). Dabei hängt soziale Kontrolle insbesondere davon ab, ob es eine gemeinsame Vorstellung und Erwartung darüber gibt, welches Verhalten und welche Personen zu einem Raum gehören sowie wer wie viel Verantwortung und Kontrolle über diesen Raum hat. Dieses komplexe Gefüge von Verhaltenserwartungen und -Anpassungen, auch „territorial functioning“ (Taylor und Brower 1985, S. 185) genannt, ist aufgrund der Besonderheiten des Bahnhofsviertels (hohe Fluktuation, räumliche Enge, Anonymität) anfälliger für Störungen und kann folglich schneller zu einer perzipierten Unsicherheit führen (Rölle und Flade 2004, S. 778). Dieses empirische und theoretische Vorwissen wurde im weiteren Verlauf zur forschungsleitenden Frage, weshalb die Erkenntnisse der quantitativen Befragung als eine Schärfung der analytischen Perspektive und des Erkenntnisinteresses für die weitere qualitative Erhebung verstanden werden können (Przyborski und Wohlrab-Sahr 2014, S. 30). Darauf aufbauend erfolgte ein theoretisches Sampling, also eine Fallauswahl entlang des bisher vollzogenen Forschungsprozesses und der parallel erfolgenden Analyse (Strübing 2014, S. 29). Nach ersten Interviews kristallisierten sich die Gewerbetreibenden im Münchner Bahnhofsviertel als wesentliche Beschwerdeführende und die „Tagelöhnerbörse“ als ein immer wieder genannter „Beschwerde-Hotspot“ heraus. Kontrastiert wurde die Perspektive der Gewerbetreibenden mit Interviews von Vertreterinnen und Vertretern sozialer Einrichtungen und Beratungsstellen, die direkt mit den „Tagelöhnern“ zusammenarbeiten.

Des Weiteren wurden in drei Feldaufenthalten in den Jahren 2018 und 2019 neben formalen Leitfadeninterviews ebenfalls ethnografische Interviews (Strübing 2013, S. 100 ff.) mit Gewerbetreibenden, Kunden und Anwohnenden sowie ethnografische Beobachtungen (ebd., S. 53 ff.) im Bahnhofsviertel durchgeführt, die in Feldprotokollen verschriftlicht wurden. Von zentraler Bedeutung waren auch die Gespräche, die auf Türkisch stattfanden, da die „Tagelöhner“ nicht der deutschen Sprache mächtig waren. Es wurden folglich ausschließlich türkischsprachige „Tagelöhner“ zu ihrer Lebenssituation im Münchner Bahnhofsviertel befragt, die – wie sich zeigte – die Mehrheit unter den „Tagelöhnern“ darstellen. Ein zentraler Zugang zum Feld waren dabei die Räumlichkeiten des von der Stadt finanzierten „Infozentrum Migration und Arbeit“. Dort befindet sich ein Beratungscafé, das Beratungsdienste anbietet und von den häufig auf der Straße lebenden „Tagelöhnern“ vor allem als Rückzugsort genutzt wird. Hier wurden Gruppengespräche (mit etwa fünf bis sechs Personen) geführt; ebenfalls wurden Gespräche auf der „Tagelöhnerbörse“ Ecke Goethe‑/Landwehrstraße geführt.

Die Interviewtranskripte und Feldprotokolle wurden mithilfe der qualitativen Inhaltsanalyse nach Mayring (2015) ausgewertet. Die qualitative Inhaltsanalyse, insbesondere die Technik der Strukturierung, wurde anderen Auswertungsmethoden vorgezogen, da das erläuterte empirische und theoretische Vorwissen die Erarbeitung eines Kategoriensystems erlaubte. In diesem System wurden diejenigen Aspekte festgelegt, die für die Auswertung des Materials in Hinblick auf das Erkenntnisinteresse relevant erschienen. Die zugrunde gelegten Kategorien sollten dabei Aufschluss darüber geben, welche Vorstellungen im Bahnhofsviertel über den gemeinsamen sozialen Raum bestehen, welche Personengruppen und Verhaltensweisen inkludiert bzw. exkludiert werden und welche Legitimationen herangezogen werden, um diesen sozialen und räumlichen Ausschluss zu begründen. Anhand der im Rahmen der Inhaltsanalyse analysierten Kategorien werden in diesem Beitrag Ausschnitte der Ergebnisse hinsichtlich des zentralen Erkenntnisinteresses präsentiert. Von besonderer Bedeutung sind hierbei Ethnisierungs- und Grenzziehungsprozesse, auf die in Abschn. 5 näher eingegangen wird. Zunächst erfolgt eine Beschreibung der Situation der „Tagelöhner“ im Münchner Bahnhofsviertel.

## Mitten im südlichen Münchner Bahnhofsviertel

Die Münchner Medienberichterstattung erzeugt ein eindimensionales Bild über den „Tagelöhnermarkt“ in der Nähe des Hauptbahnhofs (Riedner 2018, S. 10). Häufig ist die Rede von einem Dutzend bulgarischer Männer, die an der Ecke Goethe‑/Landwehrstraße im Münchner Bahnhofsviertel, dem despektierlich genannten „Arbeiterstrich“, Tag ein Tag aus darauf warten, dass sie jemand für Arbeit anheuert.^4^ Allerdings ist schon diese räumliche und zeitliche Eingrenzung trügerisch, denn entsprechend den Beobachtungen und Gesprächen beschränkt sich der „Tagelöhnermarkt“ weder ausschließlich auf die Ecke Goethe‑/Landwehrstraße, noch halten sich die Personen dort durchgehend auf.

Im Laufe der 2000er Jahren bildete sich im südlichen Bahnhofsviertel ein „Tagelöhnermarkt“ aus bulgarischen Arbeitern, die mehrheitlich aus der Region und Kleinstadt Pasardschik, einem verarmten Landstrich in Zentralbulgarien, stammen (vgl. Abschn. 3.1). Bereits frühmorgens bieten sie ihre Arbeitskraft für etwa neun Euro Stundenlohn an (Landeshauptstadt München 2019, S. 19) und verrichten überwiegend einfache Tätigkeiten im Bau‑, Reinigungs- und Gastronomiegewerbe im Stadtgebiet und Umland (Riedner 2019, S. 64). Über den Tag verteilt treffen sich die „Tagelöhner“ insbesondere an der bereits genannten engen und belebten Kreuzung, aber auch in anderen Straßenzügen des südlichen Bahnhofsviertels, vor den vielzähligen türkischen Geschäften und Restaurants. Zum von ihnen geschilderten Alltag gehören Polizeikontrollen im Bahnhofsumfeld und mitunter auch Razzien des Zolls. Seit Juli 2018 ist mit dem Kommunalen Außendienst (KAD) der Stadt München ein weiterer Sicherheitsakteur hinzugekommen, zu dessen Aufgaben die Verfolgung von Ordnungswidrigkeiten gehört (Haverkamp et al. 2018, S. 26).

Mit dem ausbezahlten Lohn bestreiten viele „Tagelöhner“ den Lebensunterhalt für ihre daheim bleibenden Familien, was ihnen laut ihren Aussagen zu einem bescheidenen Wohlstand verhelfen kann. Den Gesprächen zufolge fehlt ihnen in München oft das Geld für eine Bleibe, so dass sie entweder draußen oder im mittlerweile ganzjährigen Übernachtungsschutz der Stadt München in Mehrbettzimmern nächtigen.^5^ In sozialen Belangen erhielten die Arbeitenden erstmals im Jahr 2010 Unterstützung von der Initiative Zivilcourage, die ihnen zweimal wöchentlich einen vor der Witterung geschützten Treffpunkt zur Verfügung stellte (Initiative Zivilcourage München 2016). Zudem begann die Initiative Ende 2015 ebenfalls die Räumlichkeiten des Beratungscafés des „Infozentrum Migration und Arbeit“ wöchentlich zu nutzen (ebd., S. 114 f.). Das Beratungscafé dient mit professioneller Beratung und Informations- und Qualifizierungsangeboten als zentrale Anlaufstelle für EU-Angehörige in prekären Beschäftigungsverhältnissen.^6^ Unterstützung ist vor allem dann nötig, wenn Arbeitgebende den Lohn gänzlich oder in Teilen vorenthalten und noch dazu ihre Sozialversicherungspflicht ignorieren. Ohne Krankenversicherung sind die Betroffenen auf die medizinischen Dienste sozialer Einrichtungen angewiesen.

Mit der diskursiven Figur des „Tagelöhners“ verbindet sich demgemäß implizit die Vorstellung eines kurzfristigen illegalen Beschäftigungsverhältnisses. Tatsächlich üben viele „Tagelöhner“ jedoch nicht nur mehrere Stunden oder Tage dauernde Beschäftigungen aus, sondern gehen längerfristige und legale Arbeitsverhältnisse ein (Riedner 2019, S. 64). Oft möchten sie nach eigenen Aussagen in Deutschland nicht sesshaft werden, vielmehr verstehen sie sich als Saison- oder Wanderarbeitende, die fern der Heimat den Lebensunterhalt für ihre Angehörigen verdienen. Hier scheinen unterschiedliche Praktiken in einem Arbeitsmarkt auf, der sowohl von Autonomie als auch von vielfältiger Ausbeutung gekennzeichnet ist und sich derart kategorialen Festsetzungen entzieht.

## Ethnische Grenzziehung

Auch der Versuch der nationalen bzw. ethnischen Zuordnung erweist sich als problematisch, insbesondere, wenn man mit essentialistisch gedachten Vorstellungen von Ethnizität und Nationalität an die „Tagelöhner“ herantritt. So beschreiben sich einige der türkischsprachigen „Tagelöhner“ in ethnografischen Feldgesprächen als Türken, andere als bulgarische Mohammedaner oder gar als die „Enkel der Osmanen“. Wiederum ein anderer bezeichnete sich selbst als Cowboy und reagierte somit auf die Frage nach seiner Zugehörigkeit mit ironischer Ablehnung. Es werden ebenso viele Sprachen gesprochen, von türkisch und bulgarisch bis hin zu verschiedenen slawischen Sprachen. Man trifft sowohl auf gläubige Moslems als auch Christen, andere wiederum scheinen mit Religion überhaupt nichts zu tun zu haben. Für einen Außenstehenden ergibt sich so eine konfuse Uneindeutigkeit ihrer Zugehörigkeit. An dieser Stelle erscheint es schlüssig, danach zu fragen, warum ihre Zugehörigkeit überhaupt eine Rolle spielt. Hinweise zur Beantwortung der aufgeworfenen Fragen gibt ihre Lebenssituation in Bulgarien, denn hier lassen sich bereits Prozesse der ethnischen Marginalisierung feststellen, die zur gegenwärtigen Uneindeutigkeit der Zugehörigkeit der türkischsprachigen „Tagelöhner“ beigetragen haben.

### Übersicht zu muslimischen Minderheiten Bulgariens

Die „Tagelöhner“ im Münchner Bahnhofsviertel stammen überwiegend aus der Kleinstadt Pasardschik mit fast 70.000 Einwohnenden in Zentralbulgarien nahe des Rhodopengebirges. Im Rhodopengebirge leben vor allem im westlichen Teil Pomaken und im östlichen Teil bulgarische Türken. In Pasardschik gibt es eine größere Gemeinschaft muslimischer Roma, ebenso von Türken und eine kleinere von Pomaken, wobei Unklarheit über die jeweilige Bevölkerungsgröße der muslimischen Minderheiten in der Kleinstadt besteht. In den 1970er und 1980er-Jahren sahen sich muslimische Pomaken, Roma und Türken einem großen Assimilationsdruck der kommunistischen Regierung ausgesetzt, der bei den muslimischen Roma bereits in den 1950er-Jahren einsetzte. Im Zuge der „Wiedergeburts“-Politik wurden die Nachnamen von Pomaken und Türken mehrfach geändert: von türkischen in bulgarische Nachnamen und wieder zurück (Eminov 1997a, S. 230; Höpken 2014, S. 366). Kurz vor dem Zusammenbruch des kommunistischen Regimes kulminierte diese „Bulgarisierung“ 1989 in der Vertreibung von etwa 320.000 Türken in die Türkei.^7^

Während die Türken heutzutage als ethnische Minderheit anerkannt und mit einer eigenen Partei („Bewegung für Rechte und Freiheiten“) parlamentarisch eingebunden sind, gilt dies nicht für die Pomaken, die ebenfalls Muslime und mitunter türkischsprachig sind (Telbizova-Sack 1999).^8^ Aufgrund dessen verschwimmen bei den Pomaken die Grenzen zu den Bulgaren oder Türken in Bulgarien. Es gibt keine sprachlichen, kulturellen oder religiösen Besonderheiten, die auf eine eigenständige pomakische Kultur hinweisen. In ihrer Geschichte hat es keinen bedeutenden Selbstdefinitionsprozess gegeben (Karagiannis 2003, S. 40), vielmehr dominieren nationalistische Fremdzuschreibungen, die sich insbesondere im Zuge der nationalen Konstitution der Republiken Bulgarien und Türkei abgezeichnet haben. Die Pomaken befanden sich hierbei in einem seit über einem Jahrhundert währenden türkisch-bulgarischen Identitätskonflikt (Riedel 2005, S. 177 ff.).^9^ Auf der einen Seite wurden sie als „zwangsislamisierte Bulgaren“ angesehen (Höpken 2014, S. 366) und auf der anderen Seite werden die Pomaken als Nachfahren balkanischer oder anatolischer Turkvölker betrachtet (Çavuşoğlu 1993). Die heutige Türkei sieht sich als Schutzmacht dieser und anderer muslimischer Minderheiten in der Region, gleichwohl die Pomaken aufgrund ihrer bulgarischen Sprache von der Türkei nicht als ‚richtige‘ Türken anerkannt werden. Dabei ist die türkische Identität als eigene Abstammungs- und Sprachgemeinschaft eine Erfindung der modernen Türkei, insbesondere der Staatsideologie des Kemalismus, während zuvor im Osmanischen Reich eine konfessionelle, keine ethno-nationale Zugehörigkeit dominierte (Riedel 2005, S. 189). Somit ist sowohl die heutige türkische als auch die bulgarische Identität das Resultat einer essentialistischen Sprach- und Identitätspolitik, die darauf abzielte, ethnische Differenzen nach außen zu anderen Gruppen zu vergrößern und nach innen zu verkleinern (ebd., S. 193). Die Pomaken waren folglich von der gouvernementalen^10^ Hervorbringung der Bulgaren und der Türken maßgeblich beeinflusst, aber zugleich vom Nationalismus nicht erfasst. Aufgrund dessen gilt Pomake nach wie vor als Stigma,^11^ als ein Zeichen der nicht-vollwertigen Zugehörigkeit weder zur einen noch zur anderen ethnischen Gruppe (Karagiannis 2003, S. 42).

Die (türkischsprachigen) Roma teilen ein ähnliches Schicksal, wenn sie auch im Gegensatz zu den Pomaken als ethnische Minderheit anerkannt sind. Diese Anerkennung ändert aber nichts an der Tatsache, dass sie im Alltag massiven Diskriminierungen und rassistischen Beschimpfungen ausgesetzt sind, die in der bulgarischen Mehrheitsgesellschaft nach wie vor weit verbreitet sind (Kunz und Karagyosov 2015, S. 34). Vorstellungen von der „kriminellen Natur“ der Roma werden mit somatischen Merkmalen wie Haut- und Haarfarbe verknüpft, die eine soziale Ausgrenzung der „Fremden“ anhand solcher Stereotypen begünstigt (Matter 2015, S. 39 ff.). Wie bereits bei den Pomaken ist die Konstruktion dieser Fremdheit unter anderem auf die ethno-nationale Konstitution der bulgarischen Nation zurückzuführen. Anhand einer identitätsstiftenden und zugleich ausgrenzenden Dichotomisierung von ‚den Bulgaren‘ und ‚den anderen‘ wurde die Marginalisierung der Roma auch nach den Transformationsjahren 1989/1990 vorangetrieben, so dass die Demokratisierung Bulgariens nicht mit einer Aufwertung der Stellung der Roma einher ging (Schüler 2006, S. 10 f.). Vielmehr gehören die Roma zu den Verlierern der Transformationsjahre, die sich insbesondere in Form einer überproportionalen Verarmung, einer verstärkten sozialen und ökonomischen Marginalisierung sowie einer geringen gesellschaftlichen und politischen Partizipation äußert (ebd., S. 4). Unterschiedliche Auf- und Abwertungsprozesse lassen sich zudem historisch beobachten. So sicherte den Roma die geringe Bedeutung ethnischer Kriterien im Osmanischen Reich zumindest eine ökonomische Rolle zu, die sie mittlerweile in der bulgarischen Gesellschaft fast vollkommen verloren haben. Die Sichtbarkeit der verarmten Roma aktiviert heute vielmehr historisch verwurzelte antiziganistische Vorurteile, die zugleich ethnische Grenzziehungsprozesse und die Perzeption der Roma als homogene Ethnie, die den wirtschaftlichen Fortschritt Bulgariens verhindern, bestärken (Matter 2015, S. 131). Diese antiziganistischen Vorurteile hängen zudem mit der historischen Genese des „Zigeuner“-Bildes im 14./15. Jahrhundert zusammen, die auch hierzulande mit Assoziationen wie „fremd“, „kulturlos“, „nomadisch“, „archaisch“ sowie „kriminell“ verknüpft sind (Geiges et al. 2017, S. 39).

Schätzungen der Rroma Foundation gehen davon aus, dass heute in Bulgarien etwa 700.000 bis 900.000 Roma leben.^12^ Es wird vermutet, dass viele Roma aus Angst vor Diskriminierung und aufgrund ihres Misstrauens gegenüber jeglicher staatlicher Erfassung und Zählung ihre Roma-Zugehörigkeit nicht angeben.^13^ Hingegen können sich die Pomaken als nicht anerkannte ethnische Minderheit entweder zu den Bulgaren oder den Türken bekennen. Um eine gewisse Eigenständigkeit zu zeigen, gibt es Pomaken, die sich in mehrheitlich bulgarisch besiedelten Gebieten als Türken ausweisen und in mehrheitlich türkisch besiedelten Gebieten als Bulgaren (Eminov 1997a, S. 109). Dies ist bei den Roma anders. Die allgegenwärtige Angst vor Diskriminierung und Stigmatisierung führt eher zu einer Identifikation mit der jeweiligen Mehrheitsbevölkerung vor Ort. Im Vergleich zu anderen Regionen sind Muslime in der Region Pasardschik eine starke Minderheit und haben einen Anteil von knapp 15 %, was es dort lebenden Roma leichter machen könnte, sich zum Islam und zur türkischen Sprache zu bekennen (Eminov 1997b, S. 233). Die uneindeutige Selbstbeschreibung der „Tagelöhner“ im Münchner Bahnhofsviertel weist in zweierlei Richtungen: Einerseits könnten sie sich von ethnischen Zuschreibungen emanzipiert haben und andererseits könnten sie ihre ethnische Zugehörigkeit aus Furcht vor Diskriminierung verschleiern. Diese ethnische Uneindeutigkeit führt dazu, dass sich die „Tagelöhner“ der Zuordnung zu einer ethnischen Minderheit entziehen.

Hierin äußert sich eine erhebliche Diskrepanz zwischen Selbst- und Fremdzuschreibung vor allem bei den Roma unter den „Tagelöhnern“, denen noch mehr Verachtung entgegenschlägt. Zudem zeigt sich, dass die Zuordnung von außen seit jeher nach primordialistischen Kriterien wie Sprache und Aussehen stattfindet (Matter 2015, S. 40 f.). Dieser Primordialismus, der davon ausgeht, es gäbe ‚ursprüngliche‘ (primordiale) Beziehungen, die durch Geburt und Sozialisation gefestigt werden und zur Herausbildung von Gruppen und Ethnien beitragen, ist konstitutiv für die Fremdzuschreibung als Pomake und Roma. Dabei verdeutlicht der Blick in die historischen und soziokulturellen Umstände in Bulgarien – wie auch in anderen Ländern –, dass Grenzziehungen zwischen Gruppen von sozialen Prozessen abhängig sind.

### Theoretische Perspektive auf Ethnizität

Entgegen dieser im Alltag noch häufig dominierenden primordialen Vorstellungen wird Ethnizität seit der sozialkonstruktivistischen Wende nicht mehr als vorsoziale, qua Natur gegebene Eigenschaft, sondern als das Ergebnis sozialer Prozesse verstanden. Als Wegbereiter für diesen Perspektivwechsel gilt Frederik Barth (1969), der Ethnizität explizit in der Terminologie der Grenzziehung fasste. Danach sind Grenzziehungen zwischen Gruppen nicht mehr als Ausdruck kultureller Verschiedenheit zu verstehen, sondern als das Resultat sozialer Prozesse, die es notwendig machen, weniger die objektiven kulturellen Unterschiede, den „cultural stuff“ (ebd., S. 15), als vielmehr die Mechanismen und Prozesse der Grenzziehung (Selbst- und Fremdzuschreibung) in den Fokus zu rücken. Eine Zugehörigkeit zu einer ethnischen Gruppe ist demnach nicht etwas, das Individuen einfach per se besitzen, sondern etwas, das in Interaktion stetig hergestellt wird. Darauf aufbauend versteht Richard Jenkins unter sozialer Identität „the human capacity to know who is who“ (2008, S. 5). Soziale Identität verweist somit auf ein Wissen über die Klassifikationen der sozialen Welt und der Akteure in ihr. Dabei gründen Identitäten auf ein Zusammenspiel von Selbst- und Fremdzuschreibung und werden daher nicht einseitig konstruiert. Erst wenn mithilfe der externen Kategorisierung durch ‚die anderen‘ die eigene Identität validiert, also in Interaktion hergestellt wird, können sich soziale Identitäten herausbilden. Eine ‚Wir-Gruppe‘ wird erst durch die Abgrenzung zu einer ‚Ihr-Gruppe‘ relevant. Andreas Wimmer (2008) spricht an dieser Stelle von Grenzziehungsprozessen oder „boundary making“. Nach dem Grenzziehungsansatz ist Ethnizität ein sozialer Kategorisierungsprozess. Dieser Prozess besteht aus einem Zusammenspiel verschiedener Akteure in einer Dynamik von sozialem Ein- und Ausschluss. Grenzen sind somit das „Ergebnis politischer und symbolischer Auseinandersetzung um die Geltung sozialer Klassifikation“ (Wimmer 2010, S. 114). Hieraus ergibt sich die Frage, wie ethnische Grenzen in sozialer Interaktion reproduziert, verändert und auch aufgehoben werden.

Diese theoretische Perspektive lenkt im Kontext der „Tagelöhner“ im Münchner Bahnhofsviertel den Blick auf die Mittel des Ein- und Ausschlusses und die individuellen Handlungsspielräume. Hier treten insbesondere drei Mittel zum Vorschein, die in Grenzziehungsprozessen wirksam werden: Zum einem spielen Diskurse und Symbole eine entscheidende Rolle. Über Diskurse konstruieren sich Vorstellungen über ‚typische‘ Merkmale sowie ‚typisches‘ Verhalten. Ein weiteres Mittel sind Diskriminierung und Kriminalisierung, mit denen folgenreiche Unterscheidungen vorgenommen werden. Mit letzterem ist gemäß dem *Labeling-Approach* die Zuschreibung von deviantem Verhalten gemeint (Sack 1972; Becker 2019). Kriminalität ist demnach keine objektive Eigenschaft, die man feststellen kann, sondern ein Prozess der Etikettierung als ‚kriminell‘. Hier offenbart sich insbesondere die Definitionsmacht der Gewerbetreibenden, welche die „Tagelöhner“ gegenüber der Stadt München und der Polizei erfolgreich als Störenfriede charakterisieren, was auch in Abschn. 6 in Hinblick auf die Illegalisierung der „Tagelöhnerbörse“ diskutiert wird. Zwang und Macht stellen somit ein weiteres Mittel der Grenzziehung dar. Die Wirkmächtigkeit dieser Grenzziehungsprozesse hängt unmittelbar von den gesellschaftlichen Positionen der involvierten Personen ab (Wimmer 2010, S. 119 ff.). Auch der reziproke Prozess aus Selbst- und Fremdzuschreibung verdeutlicht, dass die Zugehörigkeit zu einer ethnischen Gruppe nicht völlig beliebig gewählt oder gestaltet werden kann (Moosmüller 2009, S. 15). Es lässt sich kaum abstreiten, dass ethnische Zugehörigkeiten durch einen dominanten Diskurs und seine politischen und sozialen Folgen geprägt und strukturiert werden, über die Individuen nicht einfach frei bestimmen können. Wie am Beispiel des türkisch-bulgarischen-Identitätskonfliktes ersichtlich wurde, haben lokale bzw. nationale Diskurse über Zugehörigkeiten einen entscheidenden Einfluss auf Zuschreibungsprozesse. In solchen Diskursen können Vorstellungen von Ethnizität entlang einer Entweder-Oder-Dichotomie zum Ausdruck kommen, die Personen dazu veranlassen, eine vermeintlich eindeutige Zuschreibung vorzunehmen (Duemmler und Dahinden 2016). Im Fall der ethnischen Minderheiten Bulgariens reicht das von der völligen Assimilation in die bulgarische Mehrheitsgesellschaft durch Konversion bis hin zur Identifikation als Türken (Karagiannis 2003, S. 48). Es handelt sich in beiden Fällen um eine Reaktion auf ethnische Marginalisierung, die auch im gegenwärtigen Kontext der „Tagelöhner“ in München relevant ist.

## „Tagelöhner“ zwischen Selbst- und Fremdzuschreibung

Die zum Teil historisch bedingte Uneindeutigkeit ihrer Zugehörigkeit hat für die „Tagelöhner“ weitreichende Folgen für die Bewältigung ihres Alltages im Bahnhofsviertel. Dieser Alltag ist größtenteils von einer Ablehnung der „Tagelöhner“ durch die überwiegend türkeistämmigen^14^ Anwohnenden und Gewerbetreibenden des Bahnhofsviertels charakterisiert. Ein Teil nimmt die „Tagelöhner“ als störend und für das Image des Viertels als schädlich wahr. Ihnen wird sozial abweichendes Verhalten zugeschrieben, indem sie mit Drogen- und Alkoholkonsum sowie mit verschiedenen Incivilities^15^ wie Urinieren und Müllentsorgung im öffentlichen Raum assoziiert werden. Einige „Tagelöhner“ sind zudem obdachlos und verbringen, in der Hoffnung auf Arbeit, die meiste Zeit auf der Straße, was zu einer zusätzlichen Stigmatisierung führt. Problematisiert wird zudem die Verdrängung der ‚normalen‘ Passantin, da die „Tagelöhner“ oft in großen Gruppen den Bürgersteig ‚besetzen‘ würden. Diese wahrgenommenen Problemlagen werden insbesondere von den lokalen Medien perpetuiert und von einigen Gewerbetreibenden wieder aufgegriffen (Riedner 2018, S. 103 ff.). In den Gesprächen und Interviews mit den türkeistämmigen Gewerbetreibenden kam insbesondere die Befürchtung zum Vorschein, dass sich daraus eine Benachteiligung der alteingesessenen Bevölkerung ergeben würde, weil die im Zusammenhang mit den „Tagelöhnern“ perzipierten Probleme auf alle im Bahnhofsviertel übertragen würden. Diese Befürchtung leitete sich vor allem von der Annahme ab, dass viele Menschen die ethnischen Unterschiede im Bahnhofsviertel nicht erkennen würden. Ein Gewerbetreibender äußerte sich hierzu wie folgt:

Das wird aber alles auch wirklich wahrgenommen als Türken. Also da wird der Kurde als Türke wahrgenommen, da wird der Araber als Türke wahrgenommen, der Bulgare, alles. Das habe ich schon mehrmals mitgekriegt.

Diesem Mangel an Anerkennung der ethnischen Heterogenität wird durch gezielte Grenzziehungen entgegengesteuert, indem auf die Unterschiede zwischen der ‚Wir-Gruppe‘, in diesem Fall der türkeistämmigen Bevölkerung, und der ‚Ihr-Gruppe‘, in diesem Fall die „Tagelöhner“, verwiesen wird. Als Distinktionsmerkmale von der türkischen Kultur werden die Herkunft der „Tagelöhner“ aus Bulgarien, ihre Multilingualität und ein ihnen attestierter Normenverfall gedeutet. Auch ihre Hautfarbe wird als symbolischer Marker der Zugehörigkeit zu einer ethnischen Minderheit herangezogen. Ein Sozialarbeiter beschrieb die „Tagelöhner“ in ihrer Wahrnehmung durch Außenstehende als „schwarze Bulgaren“, was wiederum von diesen als Selbstzuschreibung in den Feldgesprächen aufgegriffen wurde. So begründete ein „Tagelöhner“ die Tatsache, dass er in einem Supermarkt als unerwünscht aufgefasst wurde mit den Worten „nur weil ich eine dunkle Haut habe“. Die vermeintliche ‚Andersartigkeit‘ der „Tagelöhner“ wird mit einer wirkmächtigen Zuschreibung als „Çingene“ (Türkisch für ‚Zigeuner‘) oder Roma unterstrichen. Dadurch setzt ein Teil der türkeistämmigen Bevölkerung des Bahnhofsviertels dem Zuschreibungsspielraum der „Tagelöhner“ Grenzen: Klare Fremdzuschreibungen lösen die ethnische Uneindeutigkeit auf und zugleich wird eine Selbstzuordnung als Türken abgelehnt.

Neben diesen rassistischen und antiziganistischen Vorurteilen hängt die Stellung der „Tagelöhner“ als soziale Aussätzige aber auch mit ihrem medial zugeschriebenen Status als Migrantinnen und Migranten zusammen. Die Intersektionalität von ethnischer Marginalisierung und ‚Migrant-Sein‘ trägt zur stärkeren Problematisierung der Figur des „Tagelöhners“ bei, als es Roma oder Migrant allein leisten könnten. Deutlich zum Vorschein kommt das bei der Bezeichnung „Armutszuwanderung“ (Riedner 2018, S. 15), die insbesondere medial Anklang fand.^16^ Hier verschränken sich im Kontext der „Tagelöhner“ antiziganistische Rassismen („Nomadentum“, „Wandervolk“) mit kapitalistischen Leistungsideologien („Sozialschmarotzer“, „Sozialtourismus“). Neben ihrer kulturellen ‚Andersartigkeit‘ ist es ebenso ihre angebliche Inkompatibilität sowie Gefahr für den deutschen Arbeitsmarkt (Schwarzarbeit), die als Grundlage für ihre Problematisierung herangezogen werden. In der diskursiven Figur des „Tagelöhners“ vereinen sich bestehende antiziganistische Vorurteile mit „Neo-Rassismen“ (Dietze 2009, S. 24), die Ausgrenzung nicht mehr ausschließlich entlang der Kategorien „race“ und „culture“ vornehmen, sondern anhand neoliberaler Rationalitätslogiken. Es wird folglich unterschieden zwischen ‚guten‘ (produktiven, fleißigen, gebildeten) und ‚schlechten‘ (faulen, ungebildeten) Migrantinnen und Migranten. Mit Hilfe dieser Unterscheidung werden weiterhin Ausgrenzungs- und Diskriminierungsmechanismen aufrechterhalten, ohne vorsätzlich rassistische Begründungs- und Deutungsmuster zu bedienen. Der „Neo-Rassismus“ (Balibar 1992) artikuliert sich in einem Gesellschaftsverständnis, das Rassismus angeblich überwunden hat und für Diversität, Toleranz sowie Emanzipation eintritt, aber gleichzeitig vor homophoben, frauenfeindlichen Arabern oder sozialschmarotzenden Roma warnt (Riedner 2018, S. 123). Insbesondere letzteres evoziert negative Stereotypen wie „Sozialbetrüger“ oder „Schwarzarbeiter“, die der positiven Bezeichnung des „nützlichen Arbeiters“ (Stichwort Fachkräftemangel) gegenüberstehen.

Diese Zuschreibungen gehen im Bahnhofsviertel mit festen Grenzziehungen und Mechanismen des Ausschlusses einher, die zur weiteren Prekarisierung und Kriminalisierung der „Tagelöhner“ beitragen. Zugleich wird hier die Doppelleistung von Grenzziehung sichtbar. Sie äußert sich in der Gleichzeitigkeit von situationsbezogener Inklusion und Exklusion: Die „Tagelöhner“ erfahren von der türkeistämmigen Bevölkerung die (Fremd‑)Zuschreibung als bulgarisch oder als Roma und damit explizit nicht als türkisch. Durch das Exkludieren der anderen findet zugleich eine „Stabilisierung des Selbst“ (Mummendey und Simon 1997, S. 190) statt, eine Validierung der eigenen türkischen Zugehörigkeit, welche die Kehrseite der Exklusion darstellt. Die Zugehörigkeiten werden weiterhin normativ aufgeladen und mit bestimmten Bedeutungen und Vorstellungen über kulturelle Eigenschaften verknüpft, was wiederum als symbolisches Kapital dazu dient, Zugänge und Teilhabe am sozialen Raum zu ermöglichen oder zu verschließen (Goetze 2008, S. 257). Somit dient die Exklusion folglich nicht bloß der Herstellung von Zugehörigkeiten, sondern trägt auch wesentlich zur Stabilisierung der Machtverhältnisse im Bahnhofsviertel bei (Sundsbø 2014, S. 56). Die Abgrenzungstendenzen der türkeistämmigen Community gegenüber anderen Gruppierungen im Bahnhofsviertel werden verstärkt durch die als knapp empfundenen Ressourcen und einen Verlust der Vormachtstellung im Viertel:

Diese Straße war mal typisch türkisch und mittlerweile ist es so, dass auch die türkischen Gewerbetragenden wegfallen und das Ganze dann in arabische Hand übergeht. […] Dieses Viertel wird dann von türkischen, den türkischstämmigen Personen nicht mehr angenommen.

In diesem Kontext identifiziert der Interviewte die „Tagelöhner“ im Gegensatz zur arabischstämmigen Bevölkerung, die über entsprechendes ökonomisches Kapital verfügt, nicht als Konkurrenten. In der komplizierten türkisch-arabischen Gemengelage gelten die „Tagelöhner“ jedoch als ein Fremdkörper. Die als unzureichend wahrgenommene Differenzierung zwischen Türken, Bulgaren und Arabern führt zu weiteren Abgrenzungstendenzen sowohl gegenüber den „Tagelöhnern“ als auch gegenüber Menschen aus dem arabischen Raum. Während die arabische Bevölkerung teilweise von den bestehenden kulturellen, sozialen und religiösen Strukturen der türkeistämmigen Eingewanderten im Bahnhofsviertel profitiert und zu einem ökonomischen Rivalen aufgestiegen ist, ist das aus der Sicht desselben Interviewten bei den „Tagelöhnern“ nicht der Fall:

Die Bulgaren, die hierherkommen, die kommen alle, weil sie kein Geld haben und sie haben keine Strukturen, wo sie sich selbst in Eigenregie etwas aufbauen können und das haben die Iraker nicht gehabt. […] Es gab ja hier schon türkische kurdische Vereine und da konnten sie von diesen gesamten vorherrschenden Infrastrukturen schon profitieren.

Hier zeigt sich, wie auf lokaler Ebene durch das Hinzukommen neuer Personengruppen immer wieder neue Machtbalancen entstehen und Positionen neu verhandelt werden (Ceylan 2012, S. 112). In diesen Etablierten-Außenseiter-Figurationen (Elias und Scotson 1993) dienen Grenzziehungen auch dazu, die eigene Stellung im Viertel zu erhalten oder zu verbessern. So wie das Selbstbild der Etabliertengruppe maßgeblich durch die „Minorität ihrer ‚besten‘ Mitglieder“ (ebd., S. 13) geprägt wird, kann die Außenwahrnehmung einer Gesamtgruppe auch von einer negativ wahrgenommenen Teilgruppe beeinflusst werden. Da die „Tagelöhner“ als sozial benachteiligte und diskriminierte Außenseiter oftmals als Projektionsfläche für negative Entwicklungen im Bahnhofsviertel herhalten müssen, werden sie explizit aus dem Kreis der Türken ausgeschlossen. Dadurch werden die im Zusammenhang mit den „Tagelöhnern“ assoziierten Probleme externalisiert, so dass die Verantwortung bei anderen liegt. Eine solche figurationssoziologische Perspektive auf Etablierte und Außenseiter im Münchner Bahnhofsviertel verdeutlicht damit die sozialen Schließungsdynamiken, vernachlässigt aber die von den „Tagelöhnern“ forcierten Versuche der sozialen Öffnung. Betrachtet man die Ethnisierungsprozesse im Münchner Bahnhofsviertel hingegen als einen dynamischen, reziproken Aushandlungsprozess, wird das Handeln nicht nur auf der Seite der ausschließenden, sondern auch auf der Seite der ausgeschlossenen Personengruppen verortet. Auf diese Weise werden bei den „Tagelöhnern“ auch verschiedene Strategien der ethnischen Grenzziehung sichtbar (Wimmer 2010, S. 115), die auch als eine Form des „Stigma-Mangagements“ (Goffman 1967, S. 133 ff.) verstanden werden können: also als Versuch, ihre Zugehörigkeit zu einer stigmatisierten ethnischen Minderheit zu verschleiern. Die eingangs beschriebenen unterschiedlichen Selbstzuschreibungen der „Tagelöhner“ führen zu flexiblen Zugehörigkeitskonstruktionen, die es ihnen erlauben, verschiedene Wissensvorräte situationsspezifisch abzurufen und kontextspezifisch einzusetzen. Auf diese Weise können sie im multiethnischen Bahnhofsviertel die Interaktion mit etablierten Migrantengruppen aufnehmen, da sie sowohl auf Arbeit als auch auf das Wohlwollen der Gewerbetreibenden und Anwohnenden angewiesen sind, die gegenüber der Stadt und der Polizei eine größere Beschwerdemacht aufweisen. Sie verweisen in Gesprächen z. B. mal auf ihre türkische, mal auf ihre slawische Zugehörigkeit, wie ein „Tagelöhner“ konkret am Beispiel eines bosnischen Arbeitgebers verdeutlichte, den er als seinen „Landsmann“ bezeichnete, nur um wenige Minuten später auch die Türken entsprechend zu bezeichnen. Diese Diskrepanz wird in den geführten Gesprächen mit einem Verweis auf eine „gleiche Kultur“ oder den Islam als gemeinsamer Glaube aufgelöst. Diese Grenzaufweichung dient primär dem Zweck, „Definitionskoalitionen“ (Dellwing 2015, S. 14) einzugehen, um einen bestehenden Konflikt – wie die Wahrnehmung der „Tagelöhner“ als deviante Personengruppe – abzuschwächen, indem auf eine gemeinsame Abstammung verwiesen und Solidarität eingefordert wird. In einigen Fällen gelingt das, in anderen Fällen trifft ihre Selbstzuschreibung als Türken jedoch auf Ablehnung. Ihrer Ablehnung verleihen die türkeistämmigen Gewerbetreibenden teilweise durch eine moralische Degradierung mit der abwertenden Bezeichnung „muslimische Zigeuner“ Ausdruck und noch mehr mit der Etikettierung als kriminell. Diesen Ausgrenzungsprozessen versuchen die „Tagelöhner“ mit einer Umdeutung der ethnischen Grenzen zu ihren Gunsten zu begegnen – Andreas Wimmer spricht hier von „Inversion“ (2010, S. 117). Die Kategorie der ausgeschlossenen und verachteten Menschen wird zum „auserwählten Volk“ (ebd.) erklärt, das der anderen Gruppe moralisch, physisch und kulturell überlegen ist. In den Gesprächen mit den „Tagelöhnern“ kam das zum Tragen, wenn sie sich selbst als die „Enkel der Osmanen“ sowie als die „wahren Türken“ und ihre Ausgrenzungserfahrungen als moralische Fehler der anderen bezeichneten. Diese Äußerungen können als Versuch verstanden werden, der Gegenseite die Definitionsmacht über das Türkischsein und über deviantes Verhalten abzusprechen. Dies gelingt den „Tagelöhnern“ aber nicht, da sie über weniger Definitionsmacht als die etablierten Türkeistämmigen verfügen. Im Umgang mit Behörden verweisen sie wiederum auf ihre bulgarische Zugehörigkeit bzw. Nationalität; nicht nur betonen sie ihre rechtliche Besserstellung als EU-Bürger gegenüber der türkischen Bevölkerung, sondern fordern diese auch ein. Es finden folglich permanente Grenzziehungen und Grenzverwischungen statt. Es handelt sich um eine Hervorhebung, Verdrängung und Verschiebung einer Differenzierungslinie zu Gunsten einer anderen, um den Aushandlungsprozess über Normen und Zugehörigkeiten zu beeinflussen.

In diesen komplexen Aushandlungsprozessen ethnischer Zugehörigkeit offenbaren sich sowohl die aktive Herstellung ethnischer Differenzierung als auch die Verknüpfung dieser Differenzen mit konkreten Deutungsmustern von abweichendem Verhalten. Verstärkt werden diese Deutungsmuster durch ein kommunales Alkoholverbot und ein bundesweites Verbot von „Tagelöhnerbörsen“.

## Illegalisierung der „Tagelöhnerbörse“

Nach der Einführung eines nächtlichen Verbots des Verzehrens und Mitführens von alkoholischen Getränken^17^ am Münchner Hauptbahnhof im Januar 2017 wurde dieses im August 2019 auf den ganzen Tag ausgeweitet.^18^ Zuwiderhandlungen hiergegen können mit einer Geldbuße geahndet werden. Die Kontrolle des Verbots obliegt der Polizei und dem KAD^19^ (s. Abschn. 2), dessen häufigster Einsatzgrund im ersten Jahr seines Bestehens alkoholbedingte Störungen waren. Diese Störungen leitet der KAD an die Allgemeine Gefahrenabwehr und an die Bußgeldstelle des Kreisverwaltungsreferats weiter, die nach rechtlicher Prüfung Bußgeldbescheide erlassen und je nach Art, Schwere und Häufigkeit von KAD und Polizei kontrollierte örtliche Aufenthalts- und Betretungsverbote anordnen.^20^

Mit diesem Alkoholverbot geraten ebenfalls „Tagelöhner“ – auch aus Unwissenheit – in Konflikt. Laut einem Sozialarbeiter hielt sich ein „Tagelöhner“ nicht an einen Platzverweis wegen eines Verstoßes gegen das Alkoholverbot, weil er kein Deutsch verstand und ihm als Analphabet die ausgehändigte schriftliche Belehrung in türkischer Sprache nicht weiterhalf; die Polizei nahm ihn dann aufgrund des Verstoßes gegen den Platzverweis über Nacht in Gewahrsam.^21^ Nach der städtischen Verordnung gilt das Alkoholverbot rund um den Hauptbahnhof, aber nicht für Restaurants und Freischankflächen in der Verbotszone und Personen, die den mitgeführten Alkohol woanders zu sich nehmen möchten. Als sozial abweichendes Verhalten wird der Alkoholkonsum im öffentlichen Raum insbesondere bei marginalisierten Gruppen deklariert. Mangels anderer Verweilmöglichkeiten sind marginalisierte Gruppen jedoch auf den Aufenthalt im öffentlichen Raum angewiesen und haben kein Geld für den Verzehr von alkoholischen Getränken in Lokalen. Für die „Tagelöhner“ dient der öffentliche Raum sowohl der Anbahnung von Arbeitsverhältnissen als auch dem Freizeitvertreib mit Freunden und Bekannten. Aus der Perspektive der vom Alkoholverbot Betroffenen wie auch von einigen Beobachtenden findet hier eine ‚Kriminalisierung‘ statt, auch wenn es sich beim unerlaubten Alkoholkonsum am Hauptbahnhof um eine bloße Ordnungsstörung in Form einer bußgeldbewehrten Ordnungswidrigkeit handelt. Allerdings treffen die Folgen die Betroffenen empfindlich: Neben einem Bußgeld für den verbotenen Alkoholkonsum bedeutet ein Platzverweis soziale Exklusion und die Ingewahrsamnahme einen kurzzeitigen Freiheitsentzug.

Der soziale Ausschluss manifestiert sich jedoch nicht nur in Aufenthaltsverboten, sondern auch im Verhältnis zu den Bewohnenden und Gewerbetreibenden im Bahnhofsviertel. Zu Veranstaltungen in der Nachbarschaft werden sie nicht eingeladen und sind dort auch nicht gern gesehene Gäste. Die Gewerbetreibenden beschweren sich bei der Stadt über deren vermutete geschäftsschädigende und sicherheitsbeeinträchtigende Präsenz. Die „Tagelöhner“ berichten von alltäglichen Schikanen und Einschüchterungsversuchen: Bei Aufenthalten vor ihren Geschäften fordern Geschäftsleute sie – auch nach einem Einkauf – nachdrücklich zum Verlassen auf oder drohen ihnen gar mit der Polizei. Die ihnen entgegenschlagende Ablehnung führen die „Tagelöhner“ auch auf ihr Aussehen zurück. Noch dazu belastet sie ihr mitunter ungepflegtes Äußeres, weil sie ihre Kleidung nirgends waschen können.^22^ Allerdings profitieren die Gewerbetreibenden im Bahnhofsviertel nicht nur von den Einkäufen der „Tagelöhner“, sondern auch von deren Arbeitskraft: Gelegentlich heuern sie diese für Gelegenheitsarbeiten an. Die „Tagelöhner“ beklagen sich über das vollständige oder teilweise Vorenthalten von Löhnen insbesondere von türkischen und kroatischen Arbeitgebenden; diese Ausbeutung und schlechte Behandlung verstehen sie nicht, als sie in ihnen ‚eigene Landsleute‘ (siehe Abschn. 4) sehen. Ein Sozialarbeiter ergänzt, dass viele Arbeitgebenden eine legale Einstellung wünschen würden, eine solche aber oft an Sprachproblemen und fehlenden Dokumenten scheitere.

Zugleich kommen illegale Beschäftigungen oft genug vor. Der Sozialleistungsmissbrauch seitens der Arbeitgebenden stellt ein gesamtgesellschaftliches Problem dar, das sich in Ausfällen in der Sozialversicherung und bei den Steuereinnahmen, Schutzrechten und Sozialleistungsansprüchen der Betroffenen sowie einem verzerrten Wettbewerb unter Unternehmen äußert (Deutscher Bundestag 2019, S. 1). Arbeitgebende, die keine Sozialversicherungsbeiträge abführen, machen sich des Vorenthaltens und Veruntreuens von Arbeitsentgelt (§ 266a Strafgesetzbuch – StGB) strafbar. Auch Saison- und Wanderarbeit unterliegt beschränkt der Steuerpflicht^23^ und bei mehr als 70 Arbeitstagen jährlich der Sozialversicherungspflicht.^24^ Etwas anderes gilt für diejenigen „Tagelöhner“, die ihren Lebensmittelpunkt in die Bundesrepublik verlegen.^25^ Eine Steuerhinterziehung kommt sowohl bei den Schwarzarbeitenden als auch den Arbeitgebenden (§ 370 Abgabenordung – AO) in Betracht. Schwarzarbeit stellt für beide Parteien eine Ordnungswidrigkeit nach § 8 des Schwarzarbeitsbekämpfungsgesetzes (SchwarzArbG)^26^ dar.

Dessen Reform im Sommer 2019 führte zu einer Verschlechterung der Arbeitsbedingungen der „Tagelöhner“, da der neu eingefügte § 5a SchwarzArbG „Tagelöhnerbörsen“ im öffentlichen Raum untersagt. Nach Abs. 1 S. 1 darf eine Person ihre Arbeitskraft nicht als „Tagelöhner“ im öffentlichen Raum aus einer Gruppe heraus in einer Weise anbieten, die geeignet ist, Schwarzarbeit oder illegale Beschäftigung zu ermöglichen; und nach Abs. 1 S. 2 ist es einer Person verboten, ein unzulässiges Anbieten der Arbeitskraft dadurch nachzufragen, dass sie ein solches Angebot einholt oder annimmt. Nach der Gesetzesbegründung ist „ein Tagelöhner im Sinne der Norm […] jemand, der nicht in einem festen Beschäftigungsverhältnis steht, sondern seine Arbeitskraft in der Regel bei wechselnden Arbeitgebern kurzfristig für einen vorübergehenden Zeitraum gegen Entgelt anbietet und nicht unständig beschäftigt ist (§ 27 Absatz 3 Nummer 1 des Dritten Buches Sozialgesetzbuch)“ (Deutscher Bundestag 2019, S. 49). Hieraus ergibt sich, dass diese Definition zu kurz greift und eben nicht Zugewanderte umfasst. Das Verbot des Anbietens und Nachfragens der Arbeitskraft wird zusätzlich auf das Ausbeutungsrisiko für Arbeitssuchende gestützt (ebd.).

Im Gesetzgebungsverfahren stieß die Neuregelung auf erhebliche Kritik, die die ersatzlose Streichung empfahl. Denn die beabsichtigte Auflösung der „Tagelöhnerbörsen“ hilft nicht den hiervon Betroffenen, denen nichts anderes übrig bleibt, als im öffentlichen Raum nach Arbeit zu suchen, um den Lebensunterhalt für ihre Familien zu sichern (KOK 2019, S. 6). Noch dazu sind bei Verstößen Platzverweise und Betretungsverbote durch die Behörden der Zollverwaltung sowie die Ahndung mit einem Bußgeld bis zu 5000 € vorgesehen (§ 5a Abs. 2, § 8 Abs. 2 Nr. 6 i. V. m. Abs. 6 SchwarzArbG). Das Verbot und dessen Durchsetzung erscheinen nicht sinnvoll im Umgang mit dem gesellschaftlichen Problem der Schwarzarbeit. Dies geht auch aus einer Antwort des Münchner Kreisverwaltungsreferats auf eine Anfrage von Stadtrats-Mitgliedern hervor: „Erfahrungsgemäß sind südosteuropäische Tagelöhner fast ausschließlich in Wirtschaftsbereichen tätig, die der Sofortmeldepflicht unterliegen. Insofern ist die Feststellung und ggf. Ahndung des Beschäftigungsverhältnisses direkt am Arbeitsplatz als effektive Maßnahme angezeigt und trifft zudem den Arbeitgeber in dessen Verantwortung“ (Landeshauptstadt München 2019, S. 19). Erfolgversprechender sind also mehr Kontrollen bei den Arbeitgebenden.

Trotz des Verbots von Arbeiterbörsen ist der bloße Aufenthalt von Personen(-gruppen) im öffentlichen Raum erlaubt, da dieser einem jeden Individuum grundsätzlich offensteht (Haverkamp 2014, S. 10). Örtliche Gewerbetreibende sehen in den „Tagelöhnern“ Eindringlinge, die zwar einen Beitrag zu ihrem Profit leisten, jedoch schreiben sie ihnen einen schädigenden Einfluss auf ihre Geschäftsinteressen zu und heften ihnen dank ihrer Wirkmacht erfolgreich das Etikett des „Störenfrieds“ an (Riedner 2018, S. 117). Ein negatives Image haben die „Tagelöhner“ nicht nur in München, sondern überall dort, wo es im Bundesgebiet Arbeitermärkte gibt. Beredter Ausdruck hiervon ist die Einfügung des Verbots der „Tagelöhnerbörsen“ im Schwarzarbeitsbekämpfungsgesetz. Seit Inkrafttreten des Verbots führen die Behörden der Zollverwaltung vermehrt Kontrollen im südlichen Münchner Bahnhofsviertel durch (Landeshauptstadt München 2019, S. 19 f.). In der behördlichen Wahrnehmung hat sich vielfach das Narrativ der Arbeiterbörse als Ordnungsstörung^27^ etabliert. Entlarvend ist hier die fehlgehende Verwendung von brachialen Begriffen der Grenzzolldirektion im Umgang mit den Arbeitssuchenden: „Die *Bekämpfung von Tagelöhnern* [eigene Hervorhebung] im öffentlichen Raum, bedarf ähnlich wie andere in der öffentlichen Wahrnehmbarkeit stattfindenden Zuwiderhandlungen […] eines allumfassenden kriminologischen und damit behördenübergreifenden Lösungsansatzes“ (ebd., S. 19 f.). In den vergangenen Jahren lassen sich zwei widersprüchliche Entwicklungen ausmachen. Einerseits erfolgt ein Ausbau der sozialen Hilfsangebote für die bulgarischen „Tagelöhner“ und andererseits lässt sich ein wachsender Verdrängungsdruck durch Illegalisierung und damit einhergehend eine stärkere Stigmatisierung und Diskriminierung beobachten.

## Schlussbemerkungen

Nach wie vor ist der rassistische Spruch „Nicht jeder Dieb ist ein Zigeuner, aber jeder Zigeuner ist ein Dieb“ verbreitet. Die Romvölker, zu denen die „Tagelöhner“ teilweise gezählt werden, sind eine ethnische Minderheit, die überall in Europa seit Jahrhunderten auf Hass stößt und unter Verfolgung leidet (Bogdal 2011). Grundlage hierfür ist ein Entstehungsmythos, der sich losgelöst von den Betroffenen entwickelte und dessen historische Nachwirkungen immer noch deren Stellung als soziale Aussätzige bestimmt.

Das Ziel des Beitrages war es, ausgehend von der Frage, wie sich Nachbarschaft im Bahnhofsviertel gestaltet, die ethnischen Grenzziehungsprozesse im Münchner Bahnhofsviertel nachzuzeichnen. Hier offenbaren sich insbesondere Abgrenzungsmechanismen der etablierten Türkeistämmigen, die mit dem Mittel der Diskriminierung und Kriminalisierung Ausdruck finden. Die Gegenreaktion der „Tagelöhner“ besteht aus dem Versuch, die existierenden Grenzen umzudeuten. Daraus ergeben sich zugleich zwei interessante Einblicke: Erstens kann von Kriminalität nur perspektivisch gesprochen werden (Dollinger 2010, S. 111). Während die Präsenz der „Tagelöhner“ im Bahnhofsviertel von den Gewerbetreibenden als unerwünscht sowie ihr Verhalten als abweichend und in vielen Fällen pauschal als kriminell aufgefasst wird, sehen sich die „Tagelöhner“ als Opfer von Ausgrenzungs- sowie Diskriminierungsprozessen. Kriminalität ist ein „Ringen um Sinnanschlüsse“ (ebd.), Diskurse und Ethnisierungen helfen dabei, an bestimmte Bedeutungen anzuschließen. Zweitens wird verdeutlicht, dass sowohl Kriminalität als auch Ethnizität als artikulatorische Praxis verstanden werden können und auf der Setzung von Differenzen beruhen. Indem die „Tagelöhner“ trotz ihrer Türkischkenntnisse, ihres islamischen Glaubens und ihrer Selbstzuschreibung nicht als Türken wahrgenommen werden, wird Fremdheit erst aktiv hergestellt. Als weitere Aspekte wurden antiziganistische Vorurteile und Neo-Rassismen diskutiert, während die Illegalisierung der „Tagelöhnerbörse“ zur weiteren Legitimierung der sozialen Ausgrenzung der „Tagelöhner“ und ihrer Stigmatisierung als kriminell herausgearbeitet wurde. Es sollte gezeigt werden, dass die anfangs aufgeworfene Frage nach der Rolle ihrer Zugehörigkeit als eine Form des Stigma-Managements verstanden werden kann: Indem sie Uneindeutigkeit schaffen und wieder auflösen, versuchen sie ihre Zugehörigkeit zu einer ethnischen Minderheit, die in Bulgarien wie auch in Deutschland Diskriminierungserfahrungen ausgesetzt ist, zu verschleiern und zugleich Beziehungen mit den etablierten Migrantengruppen, speziell den Türkeistämmigen, einzugehen. Während Samim Akgönül in diesem Zusammenhang unter den muslimischen Minderheiten im Balkan eine „confusion of identity“ (Akgönül 2002, S. 145) konstatiert, offenbart sich hinter der teils historisch bedingten Konfusion auch eine Strategie des Umgangs mit ethnischer Marginalisierung. Uneindeutigkeit und multiple Identitätsorientierungen ermöglichen den „Tagelöhnern“, flexible Handlungsmöglichkeiten aufrechtzuerhalten. Sie tragen aber zugleich zu ihrer weiteren Ausgrenzung bei, die unter dem Gesichtspunkt der Aushandlung von Teilhabe und Zugang im städtischen Raum betrachtet werden muss.

Aus kommunalpräventiver Sicht erlaubt diese Perspektive, die Illegalisierung des „Tagelöhnermarktes“ und die Forderung nach mehr Sozialkontrolle, die auf den Ausschluss von marginalisierten Personengruppen aus dem öffentlichen Raum abzielen, kritisch einzuordnen. Denn abweichendes Verhalten und Kriminalität stellen „die Konsequenz eines Interaktionsprozesses zwischen Menschen“ (Becker 2019, S. 135) dar. Die Kriminalisierung der „Tagelöhner“ kann somit, wie im Beitrag argumentiert wurde, im Lichte sozialer Ausschließungsprozesse im hart umkämpften Münchner Bahnhofsviertel betrachtet werden. Verschärfende Maßnahmen und eine Ausweitung der Sozialkontrolle können folglich die Kriminalisierung der „Tagelöhner“ verstärken, statt sie abzumindern. Außerdem werden durch wirkmächtige Zuschreibungen als ‚Kriminelle‘ oder ‚Störenfriede‘ ihre weiteren Integrations- und Teilhabechancen im Münchner Bahnhofsviertel abgeschwächt. In diesen Zuschreibungsprozessen offenbart sich zudem das Zusammenspiel von Ethnisierung und Kriminalisierung: Kriminalität und Devianz werden durch unterschiedliche Sinnzuschreibungen hervorgebracht und ethnische Zugehörigkeiten stellen einen von vielen Wirkmechanismen dar, mit deren Hilfe diese Bedeutungszuweisungen hergestellt werden. Dieser höchst kontingente Prozess wird auf der Interaktionsebene, wie anhand des empirischen Materials erkennbar wurde, im Rückgriff auf Devianz- und Normativitätsvorstellungen sowie durch ethnische Grenzziehungen bestimmt. Die in diesem Beitrag diskutierte ethnische Uneindeutigkeit der „Tagelöhner“ trifft folglich auch auf die Bezeichnung ‚deviant‘ zu, die erst in der sozialen Interaktion mit Bedeutung aufgeladen wird. Die im Münchner Bahnhofsviertel involvierten Akteure bringen ihre unterschiedlichen Definitionen ein, handeln nach ihnen und treffen dann auf die Reaktionen der anderen: So werden aus migrantischen Arbeitssuchenden bulgarische „Tagelöhner“, aus einer türkischsprachigen Roma-Minderheit die „Enkel der Osmanen“; alle Definitionen sind jeweils darum bemüht, den Ein- bzw. Ausgrenzungsprozess entsprechend zu beeinflussen. Die Implementierung weiterer restriktiver Maßnahmen und die Änderung der Gesetzeslage tragen somit zur Verfestigung einer von vielen sozialen Definitionen und damit zum sozialen Ausschluss einer marginalisierten Gruppe bei.

## Footnotes

1 Es wird die männliche Form verwendet, weil sowohl im medialen als auch wissenschaftlichen Diskurs davon ausgegangen wird, dass überwiegend Männer im Münchner Bahnhofsviertel als „Tagelöhner“ tätig sind. Da es sich um einen stigmatisierenden und diskriminierenden Begriff handelt, der im Untersuchungskontext allerdings von verschiedenen Personen so genutzt wird, setzen wir den Begriff in Anführungszeichen. Wir verzichten nicht auf den Begriff, weil die Begriffe „Arbeitende“ und „Arbeitssuchende“ zu allgemein sind.

2 Weitere Informationen zu SiBa sind der Projektseite zu entnehmen. https://uni-tuebingen.de/fakultaeten/juristische-fakultaet/lehrstuehle-und-personen/lehrstuehle/lehrstuehle-strafrecht/stiftungsprofessur/haverkamp-rita/projekte/siba/. Zugegriffen: 7. Mai 2020.

3 Allerdings ist zu beachten, dass die Stichproben aufgrund der unterschiedlichen Größenverhältnisse zahlenmäßig erheblich voneinander abweichen: Während im Bahnhofsviertel lediglich 93 Befragte antworteten, waren dies für die anderen ausgewählten Stadtteile 2103.

4 Anschaulich zum Beispiel in dem Artikel *Die Tagelöhner von der Landwehrstraße* der Bayrischen Staatszeitung. https://www.bayerische-staatszeitung.de/staatszeitung/leben-in-bayern/detailansicht-leben-in-bayern/artikel/die-tageloehner-von-der-landwehrstrasse.html#topPosition. Zugegriffen: 7. Mai 2020.

5 Aus den Gesprächen mit den „Tagelöhnern“ ergab sich, dass die mitunter divergierenden Arbeitszeiten und Öffnungszeiten eine Übernachtung im Übernachtungsschutz unmöglich machen.

6 Vgl. https://www.awo-muenchen.de/migration/migrations-und-integrationsberatung/infozentrum-migration-und-arbeit/projektbeschreibung. Zugegriffen: 7. Mai 2020.

7 Vgl. https://www.nzz.ch/international/wie-bulgarien-1989-versuchte-seine-tuerken-loszuwerden-ld.1504266. Zugegriffen: 10. Mai 2020.

8 Im Gegensatz zu den Türken und Roma zählen die Pomaken nicht als anerkannte Minderheit in Bulgarien, weshalb sie in der letzten Volkszählung von 2011 nicht erfasst wurden. Schätzungen gehen jedoch von ca. 200.000 bis 250.000 Personen aus (Arndt 2013, S. 8).

9 Da die Pomaken auf beiden, der bulgarischen und griechischen Seite der Rhodopen leben, spielt Griechenland in diesem Identitätskonflikt ebenfalls eine Rolle. Im Gegensatz zu Bulgarien und der Türkei hebt Griechenland die Eigenständigkeit der pomakischen Sprache hervor. Dies muss allerdings einerseits im Lichte gegenwärtiger Bemühungen, die fortschreitende Assimilation der Pomaken in die türkische Minderheit des Landes aufzuhalten und ihnen eine unabhängige sprachliche Identität zu geben, verstanden werden (Riedel 2005, S. 159). Andererseits spielen auch vergangene Bestrebungen eine Rolle, die Pomaken nicht als bulgarischsprachige Minderheit zu definieren, um potenziellen bulgarischen Ansprüchen auf griechisches Territorium zuvorzukommen (Karagiannis 2003, S. 38).

10 Laut der Gouvernementalitätsperspektive entwickelt sich staatliche Machtausübung erst im Prozess der Festlegung von Identität, wodurch „die Bevölkerung als Datum, als Feld der Intervention und als Objekt gouvernementaler Techniken“ (Foucault 1991, S. 102) hervorgebracht wird.

11 Die Bezeichnung „Pomak“ leitet sich von den Worten „pomagač“ (Helfer der osmanischen Armee), „pomamil se“ (sich anlocken, belügen, verführen, täuschen lassen), „pomjatam se“ (sich umdrehen, sich umwenden, leugnen), „pomǎtschen“ (gezwungen, geplagt) und „poturnak“ (arm, unwissend) ab und ist somit deutlich negativ konnotiert (Telbizova-Sack 1999, S. 57).

12 Vgl. https://rroma.org/de/the-rroma/rroma-population/. Zugegriffen: 7. Mai 2020.

13 So weist das Ergebnis der letzten Volkszählung von 2011 nur 325.000 Roma aus. Vgl. https://www.nsi.bg/census2011/PDOCS2/Census2011final_en.pdf. Zugegriffen: 7. Mai 2020.

14 Von türkeistämmig ist die Rede, wenn Personengruppen gemeint sind, die entweder selbst oder deren Vorfahren aus der Türkei stammen und die daher türkische Staatsangehörige sind/waren. Türkischstämmig meint hingegen eine Person, die sich selbst, unabhängig von ihrer Herkunft und Staatsangehörigkeit, aufgrund ihrer wahrgenommenen Abstammung als türkisch bezeichnet.

15 Unter Incivilities werden subjektive Störungen der sozialen und normativen Ordnung verstanden. Als „Verletzungen gemeinschaftlich geteilter Standards“ (Lüdemann 2006, S. 288) können Incivilities auch als Signal der Erosion anerkannter Werte und sozialer Normen interpretiert werden und damit stärker in ihrer negativen Wirkung sein als bspw. Kriminalität.

16 Vgl. https://www.focus.de/panorama/welt/tid-34337/armutszuwanderung-in-muenchen-der-arbeiterstrich-schaedigt-unser-geschaeft_aid_1140034.html. Zugegriffen: 30. April 2020.

17 Verordnung der Landeshauptstadt München über das Verbot des Verzehrs und des Mitführens alkoholischer Getränke auf öffentlichen Flächen im Bereich des Hauptbahnhofes.

18 Aufgrund einer Gesetzesänderung im Mai 2018, nach der Art. 30 Abs. 1 S. 2 des bayerischen Landesstraf- und Verordnungsgesetzes den Erlass einer Gemeindeverordnung über ein ganztägiges Alkoholverbot an bestimmten öffentlichen Flächen zulässt, wenn Anhaltspunkte für die regelmäßige Begehung von Ordnungswidrigkeiten und Straftaten infolge übermäßigen Alkoholkonsums vorliegen. Allerdings ist eine solche Verordnung nach Art. 30 Abs. 1 S. 2 des Gesetzes längstens auf vier Jahre zu befristen. Gegenwärtig läuft die Verordnung am 20.01.2021 aus; in der neuen Koalitionsvereinbarung für die Münchner Stadtratsperiode 2020 bis 2026 steht das ganztägige Alkoholverbot auf dem Prüfstand (Koalitionsvereinbarung 2020, S. 34).

19 In der neuen Koalitionsvereinbarung für die Münchner Stadtratsperiode 2020 bis 2026 wird der im Juli 2018 eingeführte KAD reformiert, wobei der Fokus auf Sicherheitsdienstleistungen für städtische Gebäude liegen soll (Koalitionsvereinbarung 2020, S. 34).

20 Vgl. Rathaus Umschau 143/2019. https://ru.muenchen.de/2019/143/Alkoholverbot-am-Hauptbahnhof-ab-August-rund-um-die-Uhr-86223. Zugegriffen: 7. Mai 2020.

21 Auf Grundlage von Art. 17 Abs. 1 Nr. 4 des Gesetzes über die Aufgaben und Befugnisse der Bayerischen Staatlichen Polizei (Polizeiaufgabengesetz – PAG).

22 Im Übernachtungsschutz entsorgt das Sicherheitspersonal notdürftig im Waschbecken gereinigte und zum Trocknen aufgehängte Kleidungsstücke. In Kleiderkammern von sozialen Einrichtungen versorgen sich die „Tagelöhner“ regelmäßig mit Kleidung.

23 Nach § 1 Abs. 4 Einkommensteuergesetz besteht für Personen ohne Wohnsitz oder gewöhnlichen Aufenthalt im Inland, die aber inländische Einkünfte haben, eine beschränkte Einkommensteuerpflicht.

24 Gem. § 8 Abs. 1 Nr. 2 Sozialgesetzbuch (SGB) Viertes Buch (IV); wegen der Covid-19-Pandemie erfolgte eine Erweiterung auf 115 Arbeitstage vom 01.03.2020 bis einschließlich 31.10.2020 in § 115 SGB IV.

25 Im Falle eines Hartz-IV-Bezugs, der bei den „Tagelöhnern“ nur selten gegeben sein dürfte, liegt ein Leistungsbetrug gem. § 263 StGB wegen Nichtanzeige der Einkünfte vor.

26 Gesetz zur Bekämpfung der Schwarzarbeit und illegalen Beschäftigung.

27 Hier ist auch die Rede von „social incivilities“. Im Gegensatz zu physischen Incivilities wie Graffiti oder Müll werden mit sozialen Incivilities als störend empfundene Verhaltensweisen und Personengruppen wie z. B. herumhängende Jugendliche, alkoholisierte Personen, Drogenabhängige und Obdachlose bezeichnet (Hirtenlehner et al. 2018, S. 465).
